# Discovery of novel potential KIT inhibitors for the treatment of gastrointestinal stromal tumor

**DOI:** 10.1515/biol-2021-0036

**Published:** 2021-04-03

**Authors:** Lili Jiang, Zhongmin Zhang, Zhen Wang, Yong Liu

**Affiliations:** School of Life and Pharmaceutical Sciences, Dalian University of Technology, 2 Dagong Road, Liaodongwan New District, Panjin 124221, Liaoning, China

**Keywords:** gastrointestinal stromal tumor, KIT inhibitors, pharmacophore models, molecular docking, ADMET, virtual screening

## Abstract

Numerous inhibitors of tyrosine-protein kinase KIT, a receptor tyrosine kinase, have been explored as a viable therapy for the treatment of gastrointestinal stromal tumor (GIST). However, drug resistance due to acquired mutations in KIT makes these drugs almost useless. The present study was designed to screen the novel inhibitors against the activity of the KIT mutants through pharmacophore modeling and molecular docking. The best two pharmacophore models were established using the KIT mutants’ crystal complexes and were used to screen the new compounds with possible KIT inhibitory activity against both activation loop and ATP-binding mutants. As a result, two compounds were identified as potential candidates from the virtual screening, which satisfied the potential binding capabilities, molecular modeling characteristics, and predicted absorption, distribution, metabolism, excretion, toxicity (ADMET) properties. Further molecular docking simulations showed that two compounds made strong hydrogen bond interaction with different KIT mutant proteins. Our results indicated that pharmacophore models based on the receptor–ligand complex had excellent ability to screen KIT inhibitors, and two compounds may have the potential to develop further as the future KIT inhibitors for GIST treatment.

## Graphical abstract

**Figure:**
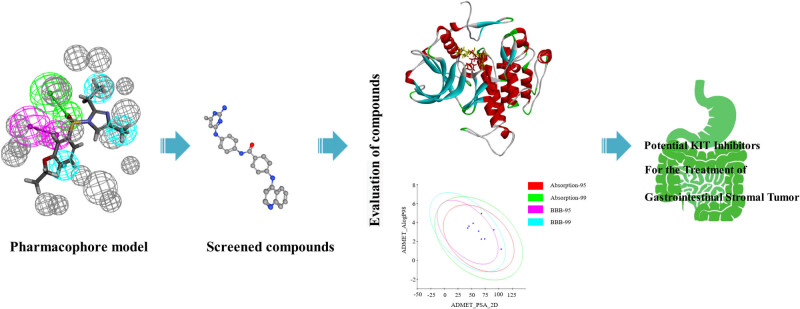

## 1 Introduction

Gastrointestinal stromal tumor (GIST) is the most common mesenchymal tumor in the gastrointestinal tract. Approximately 85–90% of GISTs are found to harbor activating mutations of KIT or platelet-derived growth factor receptor (PDGFR) [1,2]. These primary activating mutations in GIST generally occur in either KIT juxtamembrane domain (exon 11) or extracellular domain (exon 9) and rarely in the cytoplasmic ATP-binding pocket (exon 13/14) or activation loop (A-loop; exon 17) [3,4,5].

Imatinib, as a first-line therapy drug for GIST patients, has favorable effects for about 86% of KIT primary mutations (Figure S1) [6,7]. However, more than a half of imatinib-treated patients present drug resistance due to the acquired secondary KIT mutations within 2 years [8]. The majority of KIT secondary mutations affects the cytoplasmic ATP-binding pocket (exon 13/14) or A-loop (exon 17) [9]. Sunitinib, an approved second-line therapy drug for imatinib-resistant GIST patients, potently inhibits KIT of ATP-binding pocket mutants to overcome some imatinib-resistant mutants [10]. Unfortunately, sunitinib is ineffective against KIT of A-loop mutants, which accounts for about 50% of imatinib-resistance mutations [6], while ponatinib has been shown to inhibit the variants of KIT through inhibiting exon 11 primary mutants and secondary mutants of the A-loop [11]. Thus, the development of new drugs is needed to overcome resistance mutations in KIT, in particular those in ATP-binding mutants and the A-loop.

Pharmacophore model describes the spatial arrangement between a small active compound and a target protein. It can be used for virtual screening to select novel compounds that match the specified structural requirements of the binding site. The pharmacophore model is generally generated by ligand-based and structure-based methods. In the present study, we approach structure-based pharmacophore modeling, since the crystal structure of KIT protein has been released [12].

In the pursuit of overcoming resistance mutations in KIT, we developed two novel pharmacophore models using the structure-based method. Subsequently, two models were created using the known KIT inhibitors to screen for new compounds that possessed KIT inhibitory activity against both A-loop and ATP-binding pocket mutants. The new compounds were subjected to filter by ADME properties. Molecular docking of the protein–ligand complexes was employed to analyze and evaluate the affinity of the complexes, for revealing the response of protein to the binding of ligands at the atomic level.

## 2 Materials and methods

### 2.1 Dataset preparation

We selected 20 structurally diverse compounds with the reported inhibitory activity values from the literature [13,14,15,16,17,18]. The compound selection in training set was deeply considered based on the 3D quantitative structure–activity relationship generation. The respective 2D structure of the compounds with their different activity data used in the training set and test set was represented in the supporting information (Figures S2 and S3). The dataset was used for the pharmacophore validation.

### 2.2 Pharmacophore models’ generation

The pharmacophore models based on the receptor–ligand complex were built using the complex based pharmacophore (CBP) algorithm of BIOVIA Discovery Studio 2016 (DS 3.0) from two crystal structures of KIT protein. The X-ray crystal structure of complex KIT with inhibitors (PDB ID: 3G0E [7], 4U0I [11]) obtained from the RCSB Protein Data Bank (www.rcsb.org) [19]. The small molecule inhibitors, sunitinib (PDB ID: 3G0E) and ponatinib (PDB ID: 4U0I), were removed from the complexes and moved to a new window as active ligands in building the pharmacophore models. The KIT protein preparation was carried out by removing the water molecules, adding the atoms for optimizing the side-chain conformation of amino acid residues and modeling the missing loop using protein prepare of DS 3.0. Following the aforementioned steps of preparation, the protein was subjected to energy minimization by applying CHARMm minimization. Subsequently, KIT–sunitinib (PDB ID: 3G0E) and KIT–ponatinib (PDB ID: 4U0I), two protein complexes were submitted to the “Receptor–Ligand Pharmacophore Generation module” of DS 3.0 in turn. The features that have been considered for the generation of the pharmacophore models are hydrogen bond acceptor (HBA), hydrogen bond donor (HBD), hydrophobic features (HY), positive ionizable feature (P), and aromatic ring (R).

### 2.3 Pharmacophore validation

The best pharmacophore model was validated by selectivity scoring which was calculated by a method named “Rules” [20]. The method uses internal rule-based scoring function. The scoring function is based on a genetic function approximation model, which is a function of the feature set in the pharmacophore model and the feature–feature distances of different types of features.

### 2.4 Pharmacophore-based virtual screening

Small-molecule structures were downloaded from Maybridge database (http://www.maybridge.com/) and Specs database (http://www.specs.net/). These compounds were filtered by Lipinski’s Rule of Five and Veber’s drug-likeness Rules to select the ones with drug-like properties [21,22]. The receiver operating characteristic (ROC) graphs were generated and the quality values, including area under the curve as well as the enrichment factor, were calculated to validate the pharmacophore models. The pharmacophore models could be used for the following screening when their quality values were greater than 0.5. Finally, two well-validated pharmacophore models were employed as a 3D query to screen the rest of the small molecules, about 137,932 compounds.

### 2.5 ADMET prediction

ADMET means absorption, distribution, metabolism, excretion, and toxicity. The protocol uses the quantitative structure-activity relationship (QSAR) models to estimate a range of ADMET-related properties for small molecules, including aqueous solubility, blood–brain barrier penetration (BBB), cytochrome P450 2D6 inhibition, hepatotoxicity, human intestinal absorption, and plasma protein binding [23]. ADMET prediction can take out the unfit candidates early in the discovery phase, rather than during the more costly drug development phases. In this study, ADMET prediction was done via the program of ADMET Descriptors in DS 3.0.

### 2.6 Protein homology modeling

Due to the lack of crystal structure, three-dimensional structure of KIT of ATP-binding pocket mutant was performed using homology modeling by an online server of SWISS-MODEL [24]. Briefly, the complete KIT protein sequence was obtained from NCBI (https://www.ncbi.nlm.nih.gov/protein/; Accession number: AAC50969.1). The sequence of KIT was changed according to the mutation forms of the ATP-binding pocket (V654A), which was used for homology modeling by SWISS-MODEL. The template (PDB ID: 3G0E) was manually selected based on the target sequence coverage, experimental resolution, sequence identity, and similarity after sequence alignment. The generated model was selected based on the quality estimation score and the overall structure similarity. The structure refinement of the model was achieved by energy minimization via the OpenMM molecular mechanics library [25]. The quality of the homology-modeled structure of the KIT mutant V654A was evaluated with ERRAT and PROVE programs [26,27]. In addition, the crystal structures of native KIT (PDB ID: 4U0I) and KIT secondary mutants of the A-loop (D816H) (PDB ID: 3G0F [7]) were retrieved from the RCSB Protein Data Bank (http://www1.rcsb.org/).

### 2.7 Docking computation

Docking is a method to evaluate protein–ligand interactions and binding properties in order to predict the activity of the ligand molecule. In this study, we employed CDOCKER algorithm (Genetic Optimization for Ligand Docking) from BIOVIA Discovery Studio 2016 (DS 3.0), for searching the binding space and ligand conformational space. The docking used in this study was semiflexible in which the receptor proteins were rigid, but the ligands were flexible. In addition, CDOCKER in DS 3.0 used a scoring function, based on the interaction energy between receptor proteins and ligands. The three-dimensional structures of native KIT (PDB ID: 4U0I), D816H mutant KIT (PDB ID: 3G0F), and V654A mutant KIT (Homology modeling) were considered as receptors. In the protein preparation, all the water molecules and complexes bound to receptor molecule were removed, hydrogen atoms were added, and the missing atom residues were built. The binding sites of the proteins were defined based on the active sites from the PDB site records or volume occupied by the known ligand pose already in reports [28,29]. During the docking process, the top 10 ligand binding poses were saved for each ligand according to their CDOCKER energies, and the predicted binding interactions were then analyzed using the standard protocol.

## 3 Results

### 3.1 Pharmacophore models’ generation

**Figure 1:**
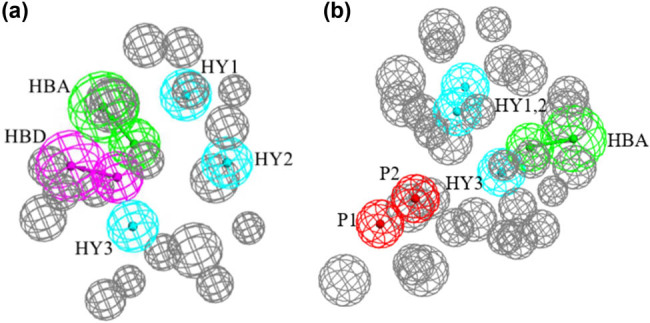
The final pharmacophore model-based KIT–sunitinib and KIT–ponatinib complexes. (a) Pharmacophore model Hypo1 for KIT–sunitinib; (b) pharmacophore model Hypo2 for KIT–ponatinib. The hypothesis features are labeled as follows: hydrogen bond donor (HBD), hydrogen bond acceptor (HBA), hydrophobic feature (HY), and positive ionizable feature (P).

**Figure 2:**
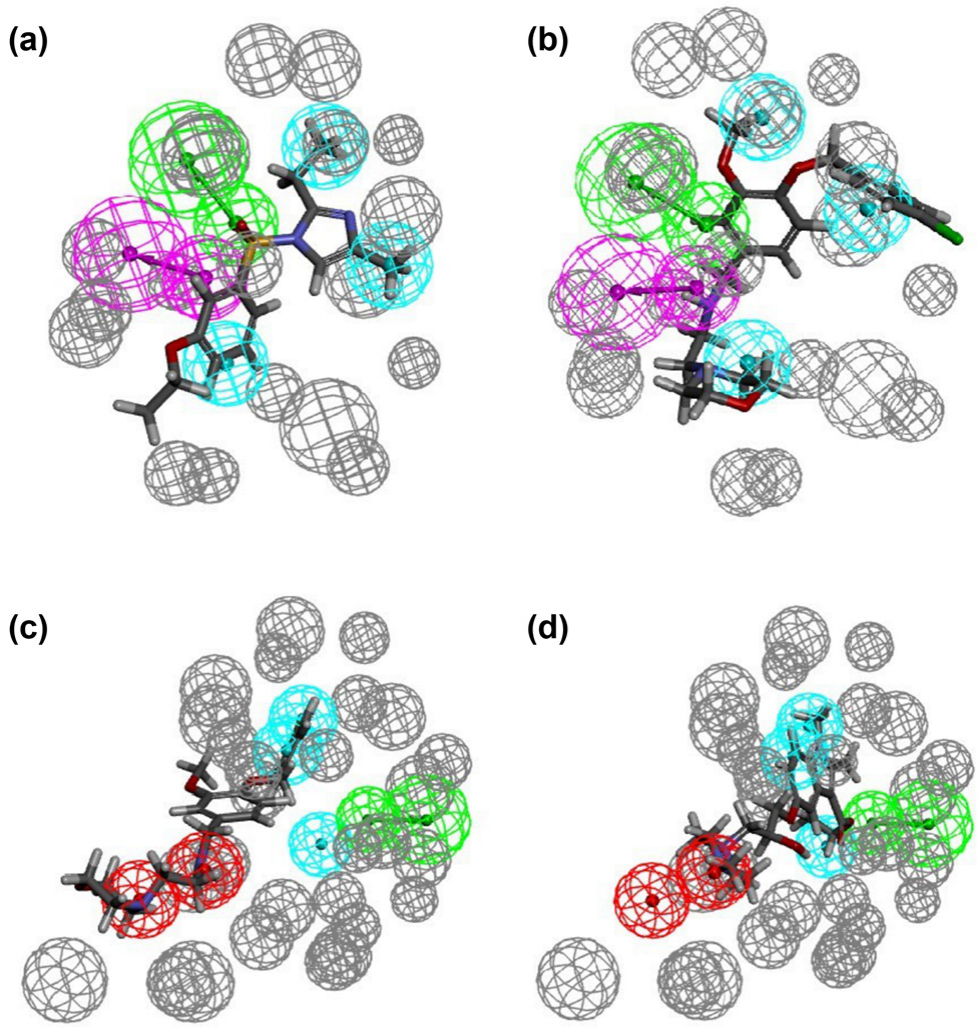
Mapping of each of the best hits to Hypo1 and Hypo2. The colors of the pharmacophore features, HBA, HBD, HY, and P are shown by green sphere, heliotrope sphere, cyan sphere, and red sphere, respectively. (a) Compound 05 is mapped with Hypo1; (b) compound 06 is mapped with Hypo1; (c) compound 05 is mapped with Hypo2; and (d) compound 06 is mapped with Hypo2.

The pharmacophore hypotheses were generated based on common features (Tables S1 and S2). As shown in Figure 1, Hypo1 and Hypo2 were picked out considering their most chemical features and the highest selectivity scoring. Hypo1, based on the KIT–sunitinib complex, consisted of one HBA, one HBD, and three hydrophobic features (HY1, HY2, and HY3). In Hypo2-based KIT–ponatinib complex three function feature sets were identified, including one HBA, three hydrophobic features (HY1, HY2, and HY3), and two positive ionizable features (P1 and P2). The generated pharmacophore models commonly contained HBA and HY features, which led to a conclusion that these two features are important for the inhibition of KIT activity. In addition, the difference between the two generated pharmacophore models (Hypo1 and Hypo2) was two positive ionizable features, which may be an effect of different KIT mutations. Figure 2, illustrates the geometrical constrains and excluded volume spheres for the features of active compounds with pharmacophore models. Figure S4 further displays the ROC graphs generated by screening the training set. The results indicated that the built pharmacophore models were more sensitive and reliable to screen the novel KIT inhibitors in the following database.

### 3.2 Pharmacophore-based virtual screening

**Figure 3:**
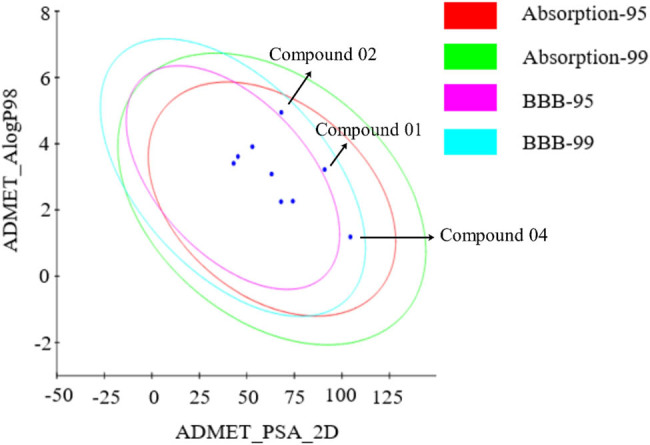
ADMET properties of the screened nine compounds. Abosorption-95 and Abosorption-99 were 95 and 99% confidence of absorption. BBB-95 and BBB-99 were 95 and 99% confidence of BBB. Almost nine compounds had excellent ADMET quality except the slightly bad aqueous solubility of three compounds (compounds 01, 02, and 04 are indicated by arrows).

Two pharmacophore models were employed to screen the database and to test the model specificity. As a result, only dozens of molecules fit all pharmacophore features of Hypo2. Because of the excellent specificity of Hypo2, Hypo1 was employed for the first screening and then Hypo2. After screening, a total of nine compounds were selected from two databases with 137,932 compounds (Figure S5). ADMET computation showed that all nine compounds have excellent ADMET quality except the slightly bad aqueous solubility of three compounds (compound 01, compound 02, and compound 04; Figure 3).

### 3.3 Model building and structure validation

Because no 3D structures for mutant KIT in ATP pocket have been reported in the PDB data bank, the homology modeling was performed to build a 3D structure of the protein [30]. The final 3D structure is shown in Figure S6a. The quality of 3D model was verified by using SWISS-MODEL server and PROCHECK program. Typically, for each residue of the model (Reported on the X-axis), the similarity to the native structure (Y-axis), showing a score above 0.6, was expected to be of high quality (Figure S6b). As shown in Figure S6c, higher QMEAN *Z* scores indicated better agreement between the model structure and experimental structures of similar size. Scores below −4.0 indicated that model’s quality was very low. The QMEAN *Z* score of predicted model was −0.18, which indicated the model’s high quality comparable to experimental structures. The Ramachandran plot for the predicted model indicated that 99.7% of residues was in the allowed regions, while only 0.3% was in the disallowed regions, confirming that the predicted model was of high quality (Figure S6d).

### 3.4 Docking result analysis

Molecule docking can make a relative accurate prediction of the interaction of small molecule with receptor. Many screening research of inhibitor-based protein structure employed mutation proteins built by homology modeling methods to dock with small molecules [31,32]. The receptor–ligand total energy (CDOCKER ENERGY) and the receptor–ligand interactional energy (CDOCKER INTERACTION ENERGY) were the main parameters of CDOCK results, which represented the stability of docking system and the interaction energy in the bonding process of receptor with ligand, respectively.

In the present study, nine compounds, which were selected based on pharmacophore models, were docked with native KIT protein and two mutation proteins. The results (Table 1) showed that all compounds (compounds 01 to 09) have excellent interaction with different proteins (Native KIT, D816H mutant KIT, and V654A mutant KIT) and indicated the high efficiency of pharmacophore models. Moreover, the docking scores were influenced by different mutation types of KIT protein. Finally, to learn more information of the interactions, compounds 05 and 06 were selected to show the 2D diagram interactions, as the potential candidates for inhibition of KIT, based on the docking results and ADMET scores.

**Table 1:** Docking result of compounds with three protein models

Protein	Compound	CDOCKER ENERGY (−kcal/mol)	CDOCKER INTERACTION ENERGY (−kcal/mol)
Native KIT	Compound 01	55.25	54.17
	Compound 02	47.52	46.49
	Compound 03	31.29	37.69
	Compound 04	30.28	51.20
	Compound 05	29.49	51.04
	Compound 06	28.03	48.68
	Compound 07	26.03	46.14
	Compound 08	17.56	40.46
	Compound 09	15.72	45.00
V654 A mutant KIT	Compound 01	57.80	56.87
	Compound 02	48.56	48.71
	Compound 04	40.14	60.53
	Compound 05	32.91	55.95
	Compound 07	31.14	52.50
	Compound 06	30.74	48.81
	Compound 03	28.53	34.54
	Compound 08	23.03	45.22
	Compound 09	20.16	54.66
D816H mutant KIT	Compound 01	57.81	58.91
	Compound 02	44.76	51.77
	Compound 04	40.14	60.53
	Compound 05	32.91	55.95
	Compound 06	29.27	47.80
	Compound 03	28.53	34.54
	Compound 09	20.16	54.66
	Compound 07	11.50	37.39
	Compound 08	−4.70	32.52

**Figure 4:**
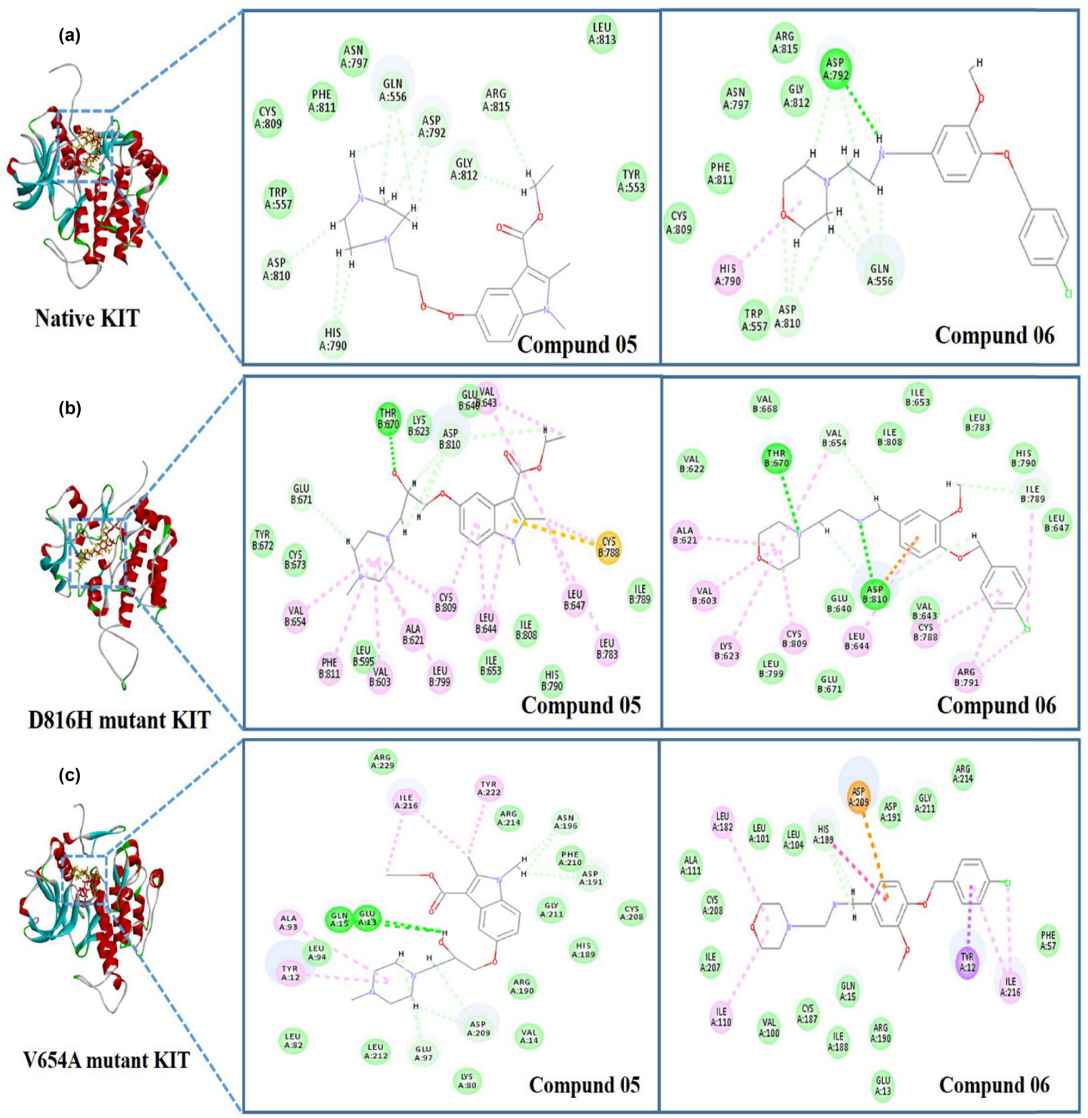
The receptor–ligand interactions of screening compounds 05 and 06 with the native KIT protein and two mutation proteins (D816H mutation KIT protein and V654A mutation KIT protein). (a) Interaction of ligands (compounds 05 and 06) with native KIT protein; (b) interaction of ligands (compounds 05 and 06) with D816H mutation KIT protein; and (c) interaction of ligands (compounds 05 and 06) with V654A mutation KIT protein. Compound 05 is red and compound 06 yellow.

The 2D diagram interactions between compounds 05 and 06, native KIT and two KIT mutants are illustrated clearly in Figure 4. The oxygen atom had strong hydrogen bond interactions with Thr670 residue in D816H, with Glu13 and Gln15 residues in V654A. More over, the benzene and nitrogen-containing heterocyclic rings formed pi-alkyl interactions with Cys809 and Leu644 residues for D816H, Ala93 and Tyr12 residues for V654A, respectively. The binding sites of interactions between compound 05, native KIT, and two KIT mutants were different, including the residues or the major force of interactions. Moreover, compound 06 mainly interacted with D816H and V654A mutant KIT via hydrogen bond interactions and pi-alkyl interactions, but the residues in the binding sites were different from that of compound 05. The diversity of the binding sites may be because of the diversity in the structure of the compounds (compounds 01 to 09) or proteins (native KIT, D816H mutant KIT, and V654A mutant KIT).

## 4 Discussion

In the past few years, several commercially available KIT inhibitors, for example, imatinib, sunitinib, and ponatinib, are under clinical investigation for GIST treatment. However, many patients are observed to experience rapid disease and present drug resistance after their treatment, which is the most common due to the acquired secondary KIT mutation [33]. The well-recognized mechanisms of acquired secondary KIT mutation include an added ATP-binding domain or the activation-loop domain of KIT [9]. Following these studies, we herein attempted to develop two novel pharmacophore models that could screen the potential candidates for KIT inhibitors with excellent effect in inhibiting two kinds of typical KIT secondary mutants. Previous study of Jiang et al. first established the three-dimensional pharmacophore model of KIT [34]. Almerico et al. also performed a pharmacophore model based on a co-crystallized compound (PDB ID: 1T46 [35]), which was a crystal structure of native KIT kinase [35]. However, their pharmacophore modeling was only used for screening KIT inhibitors without the secondary KIT mutation, which is different from our model. In addition, other studies [36,37] also developed the three-dimensional pharmacophore models of KIT, but the features of the models were different from our models developed in this study. The two pharmacophore models in this study consisted of several features, such as an HBA, an HBD, three hydrophobic features (HY1, HY2, and HY3), and two positive ionizable features (P1 and P2). These features of the pharmacophore models were representative of the characteristic of KIT mutation active site, which could be used for screening the potential candidates. So the pharmacophore models could be used as a fast and reliable tool to filter for discovering novel potential candidates for KIT inhibitor.

In addition, to get further insights into the receptor–ligand interactions between the selected compounds and two kinds of typical KIT secondary mutants, we used the pharmacophore models to screen the database. Finally, two compounds (compounds 5 and 6) were identified as active compounds, which showed excellent ADMET quality and strong interaction with two kinds of typical KIT secondary mutants involving the ATP-binding domain or the activation-loop domain. The interaction sites between compounds (compounds 5 and 6) and different proteins (native KIT, D816H mutant KIT, and V654A mutant KIT) were different, including the residues or the major interaction forces. These differences might be because of the diversity in the structure of compounds or proteins. The docking study was used to reduce false positive and identify the suitable orientation for the ligand in a protein active site as previous studies. For example, Mahadevan et al. used the molecular modeling to explain the impact of KIT mutations on imatinib resistance [38]. Hsueh et al. also introduced molecular modeling to elucidate the interaction between KIT inhibitors and mutant KIT proteins [39]. The molecular modeling showed that nilotinib had the best binding affinity for exon 11/17, which is in consistent with the *in vitro* inhibitory efficacy study on KIT mutants [39].

## 5 Conclusion

The resistance mutation in KIT is an important drawback in the clinical treatment of GIST. Hence, it is vital to consider it while exploring the new ways of treating GIST, i.e., by developing compounds that can inhibit the mutant KIT. Sunitinib and ponatinib present excellent effects for inhibiting two kinds of typical KIT secondary mutants. In the present study, the pharmacophore models were generated by using the KIT mutant crystal complexes and were employed to filter the databases. A few efficient compounds were selected. Subsequently, the potential effect was predicted by docking with the models of native KIT and two mutation proteins. The discovery of new KIT inhibitors was researched from the perspective of inhibiting different types of KIT mutants. Finally, two active compounds were identified from the virtual screening which satisfied the pharmacophore models and ADMET properties and also showed strong hydrogen bond interaction with different KIT mutant proteins. Therefore, the *in silico* screened compounds can be proposed as lead candidates and can be used for further *in vitro* and *in vivo* evaluation.
